# Effects of music on the preoperative and intraoperative anxiety through the assessment of pupil size and vital signs (blood pressure, respiratory, and pulse rates) among cataract surgery patients at UNTH-Enugu

**DOI:** 10.3389/fopht.2023.1340752

**Published:** 2024-01-15

**Authors:** Chukwubuike Obiora Ezepue, Obinna Princewill Anyatonwu, Christian Chukwuka Duru, Franklin Odini, Nkiru Zuada Nwachukwu, Chidimma Onoh, Nwamaka Nwachukwu, Chukwunonso Afam Oguonu

**Affiliations:** ^1^ Department of Ophthalmology, University of Nigeria Teaching Hospital, Ituku-Ozalla, Enugu, Nigeria; ^2^ Department of Optometry, Faculty of Medicine and Health Sciences, Abia University Uturu, Okigwe, Abia, Nigeria; ^3^ Department of Optometry, Faculty of Medicine and Health Sciences, Imo State University, Owerri, Nigeria; ^4^ Department of Community Medicine, Federal Medical Centre Umuahia, Umuahia, Abia, Nigeria; ^5^ Department of Psychiatry, University of Nigeria Teaching Hospital, Ituku-Ozalla, Enugu, Nigeria

**Keywords:** music, vitals, Nigeria, state trait anxiety questionnaire (STAI), cataract

## Abstract

**Background/Aim:**

To examine how music can impact preoperative and intraoperative anxiety via assessment of physiological markers such as pupil size, blood pressure, pulse rate, and respiratory rate.

**Methods:**

This is a randomized interventional study of individuals aged 50 years and above who were scheduled for and undergoing cataract surgery under regional anesthesia, with music (test group) randomly matched with similar individuals undergoing the same procedure but without music (control group). The surgeries were performed in the operating theater of the Department of Ophthalmology, University of Nigeria Teaching Hospital (UNTH), Ituku-Ozalla, Enugu. Using a systematic random sampling method, a total of 98 patients were grouped into two. Both groups completed the State–Trait Anxiety Inventory (STAI) questionnaire at baseline, immediately upon entrance into the preoperative room and 5 min after intervention. Relevant study indices (blood pressure, pulse, respiratory rate, and pupil diameter) were measured and recorded, and these served as baseline parameters. The STAI questionnaire was then administered. Results were analyzed using the SPSS version 20 and analysis of variance was used to compare means of variables measured at baseline, preoperative before intervention, and preoperative after intervention. Categorical variables were compared using the Chi-square test. Student’s t-test was used to analyze the continuous variables.

**Results:**

Our analysis, using the multiple linear regression, showed that music has an effect on preoperative anxiety and intraoperative anxiety by positively affecting the blood pressure, pulse rate, respiratory rate, and pupil diameter (P ≤ 0.001).

**Conclusion:**

Music reduces preoperative and intraoperative anxiety evidenced by its effect on the physiological biomarkers.

## Introduction

Globally, it is well-established that preoperative and intraoperative anxiety can have profound effects on both patients and surgical outcomes (1). According to a systematic and meta-analysis study by Abate et al. (2020), the pooled prevalence of preoperative anxiety is estimated at 48% (2). Several related epidemiological research in low- and middle-income countries found that the prevalence of preoperative anxiety varies (3–5), with Nigeria ranging from 51% to 90% (6, 7).

Anxiety is commonly characterized by restlessness, fatigue, diminished concentration, and heightened muscular tension (8). According to Liu, “Preoperative anxiety is often described as an uncomfortable, tense, and unpleasant mood before surgery, an emotional response to a potential challenge or threat to reality.” (9) The preoperative period is the period that spans from the moment the patient is informed about the procedure to be done, through the preoperative room until patient is placed on the operating table, while intraoperative period spans from the moment the patient is placed on the operating table to the moment patient is wheeled to the recovery room (Terri Goodman, 2012) (10). Preoperative anxiety is the most common form associated with a number of postoperative complications, such as delayed healing of wounds, prolonged stay in the hospital, and decreased pain threshold (11, 12).

Physiological markers such as pupil size, blood pressure, and respiratory rate are affected by anxiety. Anxiety has also been noted to have ocular manifestations such as change in intraocular pressure and pupil diameter (13, 14). Many studies have been conducted to establish the relationship between psychophysiological stress and increased intraocular pressure (14). Anxiety has been documented to activate the sympathetic nervous system, which is mediated by the hypothalamic-pituitary-adrenal axis (15). Through this pathway, there is increased circulating hormones such as cortisol, which leads to indirectly increasing the intraocular pressure by its direct effect in increasing the blood pressure (15). In anxiety states, there could be as much as 2-3-mmHg rise in intraocular pressure in normal eyes and this could be more in high-risk eyes such as patients being managed for glaucoma (15). During surgery, this increased intraocular pressure as a consequence of anxiety, combined with fluctuating pupil diameter may lead to complications intraoperatively such as iris prolapse and vitreous loss (15, 16).

Pupil diameter has also been known to be affected by stress or anxiety state (13). During small incision cataract surgery, from the onset of surgery to the placement of the intraocular lens, proper pupillary dilation is necessary and if this is not achieved, there could be iris trauma, damage to the lens zonules, posterior capsular rent with vitreous loss, and lens matter retention due to difficult lens washout (16, 17). Also immediately following intraocular lens placement, pupillary constriction is beneficial and if there is improper dilation due to anxiety, this could lead to improper placement of the lens causing iris damage intraoperatively and other complications (17). Therefore, allaying anxiety, which may lead to these intraoperative and postoperative complications is important (13).

Pharmacological products such as the benzodiazepines, either singly or in combined form for more efficacy such as the lytic cocktails comprising mainly of pethidine, chlorpromazine, and promethazine (18) have been used to reduce anxiety. These sedatives exhibit some adverse effects ranging from extrapyramidal side effects such as hallucinations to systemic hepatotoxicity, so their use have been limited (18). Music, a non-pharmacological alternative intervention and relatively cost-effective can be employed preoperatively to alleviate anxiety (19). Minichiello (2019) revealed that listening to certain types of music, particularly new-age music and classical music, can increase feelings associated with relaxation, such as peacefulness and a sense of ease (20).

In Nigeria, where preoperative anxiety and intraoperative anxiety have been documented as a prevalent concern during cataract surgery (6, 7), there is a significant gap in the understanding of how music can alleviate anxiety. While prior research has predominantly focused on the effects of pharmacological agents, there is a pressing need to explore alternative approaches. This study endeavors to address this critical knowledge gap by examining how music can impact preoperative and intraoperative anxiety via assessment of physiological markers such as pupil size, blood pressure, pulse rate, and respiratory rate. The findings of this study will provide valuable insights that can inform clinical practice and enhance the wellbeing of patients undergoing cataract surgery in Nigeria.

## Materials and methods

### Study design and setting

This study was conducted at the Department of Ophthalmology of the University of Nigeria Teaching Hospital (UNTH), Ituku-Ozalla, located 21 km from the Enugu metropolis. This was a randomized interventional study of individuals aged 50 years and above who were scheduled for and undergoing cataract surgery, with music and regional anesthesia, matched with individuals 50 years and above scheduled for and undergoing cataract surgery without music but also with regional anesthesia in the operating theater of the Department of Ophthalmology, UNTH, Ituku-Ozalla Enugu. Participants were excluded from this study if they had other types of cataracts other than age-related cataract, younger than 50 years of age, had major ocular morbidity, diagnosed with uncorrected hearing problems, and declined to participate. Patients whose age were equal to or greater than 50 years, scheduled for cataract surgery under regional anesthesia at UNTH Ituku-Ozalla, and consented to participate were enrolled in the study. A total of 98 participants were selected using a systematic random sampling method. Study participants were assigned into two groups (i.e., Group A, those that underwent surgery with music, and Group B, those that underwent surgery without music).

### Sampling technique

Patients that visited the hospital for uncomplicated cataract surgery were recruited in outpatient clinics after comprehensive clinical assessments to ensure they met the study’s eligibility criteria. Approximately 15 patients were enrolled each week and provided with detailed information about the study, the surgical procedure, and the music intervention, after which written informed consent was obtained. Enrolled individuals were randomized into two groups using a systematic sampling method, with the researcher remaining blinded to the group assignments and the randomization done by a research assistant. The patients were divided into two groups based on odd and even numbers, with odd-numbered patients (Group A) receiving surgery with music and even-numbered patients (Group B) undergoing surgery without music, while maintaining sterility with assigned earphones, and their data were meticulously recorded. This randomization was done by the research assistant, and the researcher was blinded to the groups.

### Sample size determination

The minimum sample size (n) for this study was determined using the formula for comparison between two groups/proportions. Sample size calculation was based on prevalence and the results of similar studies among preoperative anxiety studies in cataract patient (21).

Zα = the standard normal variate at 95% confidence interval if p<0.05, which equals 1.96.

Zβ = power of the study at 80% from the Z-table, which equals 0.842.

P1 = the proportion of patients who used preoperative and intraoperative music – 0.6454.23.

P2 = the proportion of patients who did not use preoperative and intraoperative music – 0.3546.23.

(P1−P2)2 = the difference in independent proportions, which equals (0.6454−0.3546)2 = 0.0846.

The minimum sample size was adjusted based on the probability of a non-response rate of 10% with the formula:

where n = minimum sample size, which equals 42 and r = 10% or 0.1.

Thus, the sample size for the two groups = 47 × 2 = 94, so n = 94.

Increasing n by 10% to leave room for errors, which involves any factor that would make any participant not show up or have his/her surgery suddenly cancelled = 94+ (10% of 94).

= 94 + 10

n = 104

### Study instrument and procedure

A pretest was carried out on six patients aged 50 years and above and scheduled for uncomplicated cataract surgery at the Enugu State University Teaching Hospital Parklane, Enugu, which is approximately a 2-h drive from the UNTH study center. State–Trait Anxiety Inventory (STAI) questionnaire was administered on the patients, and different measurements were taken at different times. This was used to determine the adequacy of the materials and methods. A pilot study was subsequently carried out on 10 patients aged 50 years and above and scheduled for uncomplicated cataract surgery at the Enugu State University Teaching Hospital Parklane, Enugu, with the help of study assistants. This was aimed at determining the feasibility of the study. Patients used in the pilot study were not included in the index study.

#### For group A: baseline

This was the routine for the test group and the control group. In the clinic, an anterior and posterior segment examination was done with the aid of a slit lamp and 90D lens, respectively, to be certain that the patient met the inclusion criteria. Rinne’s and Weber’s tests were done to ascertain patients with uncorrected hearing problems. Study participants were informed about the study´s components and objectives, and written informed consent was obtained upon recruitment in the clinic. The patients were admitted a day before the surgery, and after approximately a 30-min rest on the bed, baseline parameters were taken including blood pressure, pulse, respiratory rate, and pupil diameter, serving as objective parameters for the indirect assessment of anxiety. Blood pressure measurement was done with each patient lying down, sitting, and standing to rule out positional change in blood pressure. The pupil diameter was checked on the contralateral eye to avoid interfering with the eye to be operated on. The pupil diameter was assessed using a transparent meter rule, and the patient was given a target to look at vertically upwards while in a supine position. The STAI questionnaire was then administered. This was the subjective parameter for anxiety assessment. The patients were allowed to choose their preferred music before being transferred to the preoperative room.

#### Preoperative

In the preoperative room, the objective indices were checked and recorded again in the same manner. The STAI questionnaire was subsequently administered to the patients again. Patients were given earphones and helped to put them in place. Their preferred music was turned on and the volume was adjusted to the patient’s satisfaction. After 5 min of listening to music, the blood pressure, pulse rate, and respiratory rate were checked again and recorded. The pupil diameter was measured again on the contralateral eye and recorded, after which peribulbar anesthesia was given. The STAI questionnaire was again administered on the patients intraoperatively. Each patient was wheeled into the theater with the instrument used in measuring blood pressure and pulse still attached to the patient and the earphone still in situ, with the music still playing. The surgeon was blinded as to the group each patient belonged to. The music played for the whole duration of the surgery. At the end of surgery, as soon as the drapes were removed, the pupil diameter was measured on the contralateral eye. The music was then discontinued and each patient was wheeled out of the theater.

#### For group B

The patients in this group were processed exactly the same way and in the same order as the patients in Group A for the baseline, preoperative, and intraoperative stages. However, music was not played though the earphones were attached as with the patients in Group A.

### Data analysis

Data collected from the study were initially entered into a Microsoft Excel spreadsheet, underwent cleaning and coding, and were later imported into SPSS version 20 for analysis. Analysis included the use of tables, charts, Chi-square tests for categorical variables, Student’s t-test for continuous variables to compare pre- and post-intervention means in both groups, and ANOVA for comparing means involving more than two variables, with a significance level set at a P-value of less than 0.05.

### Ethical considerations

Ethical approval for this study was sought and obtained from the Health Research and Ethics Committee of UNTH, Ituku-Ozalla. Permission was sought from the consultants operating in the theater where the study was carried out and also from the Head of the Department of Ophthalmology. A written informed consent was obtained from the study participants. Participants were informed of their right to decline or withdraw from the study at any point without any consequences. Data collected for this research were stored in password-protected computers and the names of the participants were made anonymous.

## Results

**Table 1** shows the sociodemographic characteristics of the participants in terms of age, sex distribution, marital status, ethnicity, and religion. There was no statistically significant difference between the two groups with P-values as indicated.

**Table 1:** Socio-demographic characteristics of the study participants (n = 98).

	With music n =49 (%)	No music n=49 (%)	Total n=98 (%)	P-value
Age group				
51–60	20 (40.8)	18 (36.7)	38 (38.8)	0.590
61–70	16 (32.7)	18 (36.7)	34 (34.7)	
>70	13 (26.5)	13 (26.5)	26 (26.5)	
***Mean ± SD***	*64.27* ***±*** *7.58*	*66.14* ***±*** *9.27*		
Sex				
Male	28 (57.1)	34 (69.4)	62 (63.3)	0.102
Female	21 (42.9)	15 (30.6)	36 (36.7)	
Marital status				
Married	41 (83.7)	46 (93.9)	87 (88.8)	0.193
Not married	8 (16.3)	3 (6.1)	11 (11.2)	
Ethnicity				
Igbo Others	47 (95.9) 2 (4.1)	44 (89.8) 5 (10.2)	91 (92.9) 7 (7.1)	0.661
Religion				
Christianity Others	46 (93.9) 3 (6.1)	47 (95.9) 2 (4.1)	93 (94.9) 5 (5.1)	0.999

**Table 2** gives the baseline clinical parameters of the participants. Both groups were comparable with respect to systolic and diastolic blood pressure. Therefore, these parameters were further analyzed using multiple linear regression.

**Table 2:** Baseline clinical characteristics of the participants taken on admission into the ward.

Baseline clinical parameters	With music mean ± SD N=49	Without music mean ± SD N=49	Total	P-value
Systolic blood pressure	122.02 ± 11.62	126.18 ± 11.48	1.78	0.078
Diastolic blood pressure	78.22 ± 10.61	76.76 ± 6.49	0.83	0.41
Pulse rate	78.35 ± 3.68	73.14 ± 6.77	4.73	0.001
Respiratory rate	18.67 ± 1.89	18.86 ± 2.72	0.39	0.699
Pupil diameter	2.17 ± 0.25	2.07 ± 0.16	2.2	0.03
Anxiety score	42.67 ± 3.22	44.18 ± 2.43	2.62	0.01

**Table 3** provides the comparison of blood pressure (systolic/diastolic), pulse rate, and respiratory rate within the groups, before and after intervent*i*on. For the music group, the systolic and diastolic pressures decreased by 14.88 mmHg (95% CI =12.06–17.69) and 6.37 (95% CI =4.80–7.94). However, the systolic and diastolic blood pressure increased by 5.06 mmHg (95% CI = 2.86–7.27) and 2.65 (95 CI = 0.98–4.33) in the no-music group.

**Table 3:** Intragroup comparison of mean systolic, diastolic blood pressure, pulse rate, and respiratory rate before and after music was played for the music and no-music groups.

	With music				Without music			
	Before mean ± SD	After mean ± SD	MD	P*	Before mean ± SD	After mean ± SD	MD	P*
Systolic	141.78 ± 12.4	126.90 ± 12.1	14.88	0.001	134.02 ± 11.0	139.08 ± 11.8	-5.06	0.001
Diastolic	86.94 ± 4.5	80.57 ± 5.0	6.37	0.001	83.96 ± 5.6	86.61 ± 5.4	-2.65	0.003
Pulse rate	83.12 ± 3.0	79.20 ± 3.2	3.92	0.001	77.61 ± 7.3	79.22 ± 6.9	-1.61	0.001
Respiratory rate	22.12 ± 1.7	19.39 ± 1.8	2.73	0.001	21.53 ± 2.7	21.76 ± 2.9	-0.23	0.49

*P-values were derived using paired t-test. MD, mean difference.

**Table 4** shows the intergroup comparison of prevalence of blood pressure before and after music intervention. The blood pressure was significantly higher (51% vs. 22.4%) in the music group prior to the music intervention and the odds of having abnormal blood pressure was 3.59 times (95% CI: 1.38–9.55, p = 0.003) greater in the music group. However, following the music intervention, the blood pressure in the music group became significantly lower (14% vs. 38%) and the odds of having high blood pressure was lower 0.26 (95% CI: 0.08–7.66, p= 0.005). Conversely, the blood pressure rather increased in the group with no music intervention (22.4% →38%), p = 0.010.

**Table 4:** Effect of music on blood pressure.

	Blood pressure				
	High	Normal			
	n (%)	n (%)	χ2	OR (95% CI)	P-value
Preoperative					
With music	25 (51.0)	24 (49.0)	8.60	3.59 (1.38–9.55)	0.003
No music	11 (22.4)	38 (77.6)			
After 5 min					
With music	7 (14.3)	42 (85.7)	7.53	0.26 (0.08–7.66)	0.005
No music	19 (38.8)	30 (61.2)			

**Table 5** provides the intergroup comparison of mean systolic blood pressure, diastolic blood pressure, pulse rate, and respiratory rate between the music group and the no-music group. Apart from respiratory rate, the other parameters were significantly lower in the no-music group compared to the music group in the preoperative period. However, after the music intervention, the mean values for all the clinical measurements became significantly higher in the no-music group. Furthermore, in the no-music group, there was a linear increase for the vital signs across the different times of measurement. These observed linear trends of incremental rise with time were statistically significant for systolic blood pressure, diastolic blood pressure, pulse rate, and respiratory rate, using one way ANOVA for the repeated measure test for trend.

**Table 5:** Comparison of mean systolic, diastolic blood pressure, pulse rate, and respiratory rate between the music group and the no-music group.

With music		Without music		
Mean ± SD		Mean ± SD	T	P-value
N=49		N=49		
Preoperative clinical parameters				
Systolic	141.78 ± 12.47	134.02 ± 11.07	3.26	0.002
Diastolic	86.94 ± 4.54	83.96 ± 5.66	2.87	0.005
Pulse rate	83.12 ± 3.00	77.61 ± 7.33	4.87	0.001
Respiratory rate	22.12 ± 1.79	21.53 ± 2.71	1.28	0.205
5 min preoperative clinical parameters				
Systolic	126.90 ± 12.12	139.08 ± 11.86	5.03	0.001
Diastolic	80.57 ± 5.03	86.61 ± 5.46	5.70	0.001
Pulse rate	79.20 ± 3.27	79.22 ± 6.94	0.02	0.985
Respiratory rate	19.39 ± 1.88	21.76 ± 2.91	4.78	0.001
Intraoperative clinical parameters				
Systolic	125.55 ± 12.46	145.12 ± 13.82	7.36	0.001
Diastolic	80.41 ± 5.38	88.90 ± 5.29	7.87	0.001
Pulse rate	78.94 ± 3.26	81.06 ± 6.04	2.16	0.033
Respiratory rate	19.29 ± 1.91	22.33 ± 2.97	6.03	0.001

Multiple linear regression was fitted to determine the independent effect of music on systolic blood pressure while adjusting for the effect of differences in pre-intervention (before music). After adjusting for the differences in pre-intervention systolic blood pressure, music still had a significant effect on post- intervention systolic blood pressure (p = 0.001). The model indicates that a participant in the music group, after listening to music, on average, had systolic blood pressure 17.91 mmHg (95% CI: 14.8–21.4) lower compared to participants in the no-music group who had the same pre-intervention anxiety score (see the **Supplementary Table 6A**).

Similarly, the **Supplementary Tables 6B–D**, after adjusting for the differences in pre-intervention diastolic blood pressure, pre-intervention pulse rate, and pre-intervention respiratory rate, the music group had average post-intervention diastolic blood pressure, post-intervention pulse rate, and post-intervention respiratory rate 7.28 mmHg (95% CI = −9.29, −5.26), 4.63 (95% CI: −5.86, −3.41), and 2.76 (95% CI = −3.54–1.99) lower, respectively, compared to the no-music group (see the **Supplementary Table 6A**). Multiple logistic regression was done to determine the effect of music on the likelihood of having high blood pressure. After adjusting for the differences in pre-intervention blood pressure values, the odds of having high blood pressure were significantly greater in the no-music group, with OR = 5.16, 95% CI = 1.22–21.85, p =0.026 (see the **Supplementary Table 6B**).

**Supplementary Table 7** provides the comparative mean pupil diameter of the group with music and without music at different times during the study. The mean pupil diameter was significantly lower in the no-music group compared to the music group at baseline and the preoperative period. However, after the music intervention, the mean pupil diameter became significantly lower for the music group compared to the no-music group. Furthermore, in the no-music group, there was a significant linear increase in the mean pupil diameter across the different times of measurement.

## Discussion

The present study examined the effect of music on the level of preoperative and intraoperative anxiety in patients scheduled to undergo cataract surgery. Anxiety levels, measured using the STAI questionnaire and physiological indices (blood pressure, pulse, respiratory rate, and pupil diameter) were assessed at baseline, before, during, and after the surgery and were compared between two groups of participants, one with music intervention and the other without.

With regard to the effect on blood pressure, respiratory rate, and pulse rate, the participants that received music intervention had significantly lower values of their systolic and diastolic blood pressure and respiratory rate compared to the control group. The pulse rate of the music intervention group though lower was not significantly different from that of the control group. Camara et al. (22) and Merakou et al. (23) found that playing live classical music significantly lowered the blood pressure, respiratory rate, and pulse rate of pre-surgical patients compared to pre-exposure values (22, 24). This finding is similar to the findings by Labrague and McEnroe-Petitte (25). The result of their study involving women undergoing gynecological surgery showed that music intervention led to a significantly lower systolic and diastolic blood pressure and pulse rate. A meta-analysis of studies on music interventions for preoperative anxiety by Bradt et al. (26) also reported that music significantly reduced heart rate and diastolic blood pressure with an insignificant effect on respiratory rates (26). Another study conducted among patients undergoing abdominal surgery concluded that there was no significant difference between the anxiety level and physiological responses in two groups of patients before intervention. The findings indicate a statistically significant difference in the level of anxiety and mean blood pressure in the intervention group (p<0.05). Also, the heart and respiratory rates in the two groups of patients revealed no significant difference (27).

In our study, there was significant reduction in pupil diameter in the patients who received music intervention while those who were not exposed to music had sustained dilatation. Research has shown that pupil diameter is increased as a result of anxiety arising from anticipated distress and pain due to increased sympathetic outflow (28, 29). The reduction in pupil diameter in this study and related study (28) is therefore an indirect assessment of anxiety. It has been shown that this reduction may have been caused by a reduction in neuronal activity in the amygdala and locus coeruleus, hence the reduced pupil diameter (29). This shows that there is a likely relationship between music and pupil sizes. Also, there is a relationship between pupil size and anxiety state, which can all be influenced by music.

This study has some limitations that need to be acknowledged. Patients often struggled to comply because of survey fatigue from repeated questioning, while some had difficulty hearing the interviewer due to music-playing earphones. Conducting interviews during surgery proved distracting for both the patients and surgeons. As a result, many studies opt for the STAI questionnaire to assess anxiety before or after surgery, rather than during the procedure. Notably, the two patient groups were not age- and sex-matched, potentially introducing confounding variables. Preoperative and intraoperative anxiety present challenges in patients scheduled for and undergoing cataract surgery. There is also an effect of music on preoperative and intraoperative anxiety in patients undergoing cataract surgery at UNTH Enugu. These indirect effects can be evidenced by indirect effects of music on blood pressure, pulse rate, respiratory rate, and pupil diameter. Music has been shown by this study to be a valuable non-pharmacological agent that could be used to allay preoperative and intraoperative anxiety in such patients.

## Data availability statement

The original contributions presented in the study are included in the article/**Supplementary Material**. Further inquiries can be directed to the corresponding author.

## Ethics statement

The studies involving humans were approved by Ethical approval for this study was obtained from the Health Research and Ethics Committee of University of Nigeria Teaching Hospital, Ituku Ozalla (NHREC/05/01/2008B-FWA0002458-1RB0002323). Written informed consent was obtained from all the study participants. Participants were told of their right to decline or withdraw from the study at any point, without any consequences. Data collected for this research were stored in password-protected computers, and participant names were anonymized. Participants gave informed consent to participate in the study before taking part. The studies were conducted in accordance with the local legislation and institutional requirements. The participants provided their written informed consent to participate in this study.

## Author contributions

CE: Conceptualization, Investigation, Writing – original draft, Writing – review & editing, Formal Analysis. OA: Data curation, Software, Validation, Writing – review & editing. CD: Conceptualization, Data curation, Methodology, Resources, Validation, Writing – review & editing. FO: Conceptualization, Investigation, Methodology, Resources, Validation, Writing – review & editing. NZN: Formal Analysis, Investigation, Methodology, Validation, Writing – review & editing. NN: Conceptualization, Investigation, Methodology, Validation, Visualization, Writing – original draft, Writing – review & editing. CO: Conceptualization, Investigation, Methodology, Project administration, Resources, Supervision, Validation, Writing – review & editing. CAO: Conceptualization, Resources, Software, Validation, Writing – original draft.
