# Supplementary material for: Effects of music on the preoperative and intraoperative anxiety through the assessment of pupil size and vital signs (blood pressure, respiratory, and pulse rates) among cataract surgery patients at UNTH-Enugu
Source: Front Ophthalmol (Lausanne). 2024 Jan 15;3:1340752. doi: 10.3389/fopht.2023.1340752 (PMC11182256; doi:10.3389/fopht.2023.1340752)
Supplement: Supplementary file 1 [file Table_1.docx]

**eTable 6a: Multiple Linear Regression of post intervention systolic blood pressure**

|  | **Unstandardized**  **Coefficients** | **Standardized**  **Coefficients** |  | |
| --- | --- | --- | --- | --- |
|  | **B (95% CI)** | **Beta** | **T** | **P** |
| Music group | -17.9 (21.4 -14.8) | 0.671 | 10.15 | 0.001 |
| Pre intervention anxiety scores.  (Constant) | 0.738  4.29 | 0.681 | 10.29  0.38 | 0.001  0.699 |

| **Group Membership** | **AOR (95% CI)** | **P-Value** |
| --- | --- | --- |
| No Music | 5.16(1.22-21.85) | 0.026 |
| Music cohort |  |  |
| **Preintervention Blood pressure** | 1.26(1.14-1.40) | 0.001 |

**eTable 6b: Multiple Logistic Regression of post intervention systolic blood pressure**

**eTable 6c: Multiple Linear Regression of post intervention pulse rate**

|  | **Unstandardized**  **Coefficients** |  | **Standardized Coefficients** |  | |
| --- | --- | --- | --- | --- | --- |
|  | **B (95% CI)** |  | **Beta** | **T** | **P** |
| Music group | -4.63(-5.86, -3.41) | - 0.43 |  | -7.49 | 0.001 |
| Pre intervention pulse rate.  (Constant) | 0.83(0.74-0.94)  14.21 | 0.96 |  | 16.76  3.64 | 0.001  0.001 |

**eTable 6d: Multiple Linear Regression of post intervention respiratory rate**

|  | **Unstandardized**  **Coefficients** |  | **Standardized**  **Coefficients** |  | |
| --- | --- | --- | --- | --- | --- |
|  | **B (95% CI)** |  | **Beta** | **T** | **P** |
| Music group | -2.76(-3.54, -1.99) |  | -0.51 | -7.06 | 0.001 |
| Pre intervention respiratory rate.  (Constant) | 0.67(0.50-0.84)  7.36 |  | 0.57 | 7.83  3.95 | 0.001  0.001 |

**eTable 7: Comparison of mean pupil diameter between the group with music and without music**

| **With Music** | | **Without Music** |  |  |
| --- | --- | --- | --- | --- |
| **Mean±SD** | | **Mean±SD** | **T** | **P** |
| **N=49** | | **N=49** |  |  |
| **Pupil diameter** | |  |  |  |
| Baseline | 2.17±0.25 | 2.07±0.16 | 2.20 | 0.030 |
| Preoperative | 2.65±0.28 | 2.20±0.27 | 8.12 | 0.001 |
| 5 minutes  Preoperative | 2.23±0.28 | 2.39±0.17 | 3.37 | 0.001 |
| Intraoperative | 2.22±0.28 | 2.41±0.17 | 4.07 | 0.001 |
| *ANOVA (p-value) ** | | *F=167.36(0.001)* |  |  |

**One way ANOVA for repeated measures test for linear trend, t= independent t test*
